# Loneliness, Escapism, and Identification With Media Characters: An Exploration of the Psychological Factors Underlying Binge-Watching Tendency

**DOI:** 10.3389/fpsyg.2021.785970

**Published:** 2021-12-15

**Authors:** Alessandro Gabbiadini, Cristina Baldissarri, Roberta Rosa Valtorta, Federica Durante, Silvia Mari

**Affiliations:** Department of Psychology, Bicocca Center for Applied Psychology, Mind and Behavior Technological Center, University of Milano-Bicocca, Milan, Italy

**Keywords:** escapism, binge-watching, digital technologies problematic use, loneliness, identification

## Abstract

Nowadays, binge-watching (i.e., watching multiple episodes of a TV series in one session) has become a widespread practice of media consumption, raising concerns about its negative outcomes. Nevertheless, previous research has overlooked the underlying psychological mechanisms leading to binge-watching. In the present work, we investigated some of the psychological variables that could favor binge-watching tendencies in a sample of TV series viewers (*N* = 196). To this aim, psychological determinants of problematic digital technologies usage (i.e., feelings of loneliness), as well as some of the mechanisms related to the enjoyment of media contents (i.e., escapism and the identification with media characters), were considered as predictors of the tendency to binge-watch. Results indicated that higher feelings of loneliness were associated with higher levels of problematic digital technologies usage. Additionally, direct and indirect effects showed that only escapism – out of the four dimensions measuring the problematic use of Internet-related technologies – predicted participants’ stronger identification with media characters, which in turn promoted greater binge-watching tendencies. Overall, we suggest that binge-watching could be interpreted as a coping strategy for media escapists, who enjoy TV series as a privileged online space in which the need to escape finds its fulfillment, allowing them to manage loneliness by identifying with a fictitious character.

“*I need to stop*

*I whispered to myself*

*as I clicked the next episode*.”

Popular Internet MEME.

## Introduction

In the last decade, thanks to the emergence and growth of web streaming systems and on-demand viewing services, such as Netflix and Amazon Prime Video, watching an entire show without waiting for the release of the next episodes has become effortless. As accessing thousands of titles on online catalogs has become easier, a new behavioral phenomenon has arisen among users, who often report binge-watching; that is, watching multiple episodes of the same TV series in one session (Flayelle et al., 2019).

In the past few years, research on binge-watching has highlighted some concerns about its physical and mental health consequences. Binge-watching has been linked to negative outcomes, such as reduced social life (De Feijter et al., 2016), poorer sleep quality (Exelmans and Van den Bulck, 2017), increased sedentary lifestyle, and being overweight (see Spruance et al., 2017; Sarfraz et al., 2019; Flayelle et al., 2020). Furthermore, several works have described binge-watching as a potential addictive disorder. In fact, as noted by Flayelle et al. (2019), many people report feeling compelled to watch “just one more” episode at the end of the previous one. Accordingly, the authors suggested that binge-watching should be considered in the broader context of the contemporary digital era, in which technologies are designed to be efficient and “addictive” (Alter, 2017), fostering new behavioral patterns of overuse. The widespread diffusion of the Internet has made modern communication and entertainment technologies “irresistible” (Alter, 2017, p. 11): people feel they have to stay constantly plugged in. In this regard, recent studies have reported several forms of digital platforms’ problematic use, such as social media addiction (Andreassen et al., 2012; Orosz et al., 2016), online gaming addiction (Kuss and Griffiths, 2012), Massive multiplayer online role-playing game addiction (Kuss et al., 2012), and even “selfie” addiction (Balakrishnan and Griffiths, 2018).

However, because of its relatively new emergence, the current literature has not yet offered a clear definition of binge-watching. Some authors proposed four different types of binge-watchers (i.e., recreational TV series viewers, regulated binge-watchers, avid binge-watchers, and unregulated binge-watchers; Flayelle et al., 2020); others differentiated high, medium, and low binge-watchers based on the number of episodes, duration, and frequency of viewing sessions (Sung et al., 2018); and still others distinguished between intentional and unintentional binge-watchers (Riddle et al., 2018). Additionally, even to assess binge-watching behavior, several proposals have been made: Some authors have suggested that both the number of consecutive episodes (e.g., Schweidel and Moe, 2016) and the viewing sessions’ duration (e.g., Trouleau et al., 2016), should be considered when measuring binge-watching; others (Vaterlaus et al., 2019) define binge-watching as a problematic behavior when view sessions exceed 4 h; and still others do not identify a minimum duration (Schweidel and Moe, 2016; Sung et al., 2018; Sharma et al., 2019) or even suggest that setting a threshold represents a valueless approach (Flayelle et al., 2017).

Given this lack of consensus, for the current study, we measured binge-watching as a behavioral tendency, with the aim to deepen the knowledge on the psychological processes favoring such tendencies. Accordingly, we investigated some of the psychological variables favoring such tendencies in a sample of TV series viewers, within the theoretical framework of problematic Internet-related technologies usage. To this aim, psychological determinants of problematic use of digital technologies (i.e., loneliness) as well as some of the mechanisms related to the enjoyment of media contents (i.e., escapism and identification with media characters) were considered as predictors of the tendency to binge-watch.

## Loneliness, Escapism, and Identification with Media Characters

The Internet helps connect the world and several studies showed that it is associated with many positive outcomes, such as the maintenance of social capital (Ellison et al., 2007), the perception of greater social support (Fogel et al., 2003), and the satisfaction of the need to belong when off-line relationships are not available (Gabbiadini et al., 2020). However, most scholars agree that problematic digital technology use exists as a general social phenomenon (e.g., Chou et al., 2005), characterized by poorly controlled behaviors on digital technologies usage (Griffiths, 1998). A recent meta-analysis determined that approximately 6% of the world’s population have at least a base level of problematic use of digital technologies (Cheng and Li, 2014).

Problematic use of digital technologies has been linked to loneliness, lack of social support, and feelings of isolation (for reviews, see Tokunaga and Rains, 2010; Nowland et al., 2018; Moretta and Buodo, 2020). Digital technologies today offer countless online environments with very different characteristics. These technologies, from online video games to social media as well as streaming services critical for binge-watching-behaviors, share the same technological base: the Internet. Some authors have also argued that the excessive use of Internet-based technologies could represent a coping strategy to alleviate dysfunctional moods or to distract attention from real-life problems (e.g., Kuss and Griffiths, 2012; Kardefelt-Winther, 2014).

From a theoretical perspective, Pontes and Griffiths (2017) have argued that problematic use of Internet-related technologies is a multifaceted construct, tapping four different domains: (1) escapism, which is seeking relief from unpleasant real-life situations; (2) dysfunctional emotional coping, namely, coping styles that employ avoidance, denial, and self-blame; (3) withdrawal symptoms, impairments and dysfunctional self-regulation, that are physical and mental effects that a person experiences after reducing or stopping using online technologies; and (4) dysfunctional online-related self-control, that is the person’s ability to exert control over the use of digital technologies.

Starting from these assumptions and considering the wide variety of digital media available today, it is therefore possible to hypothesize that different digital media may satisfy different psychological needs. Crucially, coping with loneliness is a common characteristic of individuals whose binge-watching proves to be problematic (Yee, 2006; Masur et al., 2014; Gao et al., 2017; Starosta and Izydorczyk, 2020). Accordingly, some studies suggest that individuals consider binge-watching an effective way to escape from reality because it promotes transportation experiences (Wagner, 2016), thus avoiding, at least temporarily, the negative emotions caused by loneliness. Hence, it is plausible to consider escapism as the main psychological factor, among those characterizing the multifaceted construct of problematic use of Internet-related technologies (Pontes and Griffiths, 2017), triggering binge-watching behavior as a coping strategy in response to loneliness. Indeed, escapism has been defined as a psychological strategy employed by individuals to distract from real-life problems (Young et al., 2017) and has long been considered a major motivation for television viewing behavior. Back in 1962, Katz and Foulkes pointed out that for people who feel socially isolated, the perception of alienation “produces the desire to escape, a desire which the mass media are presumed to be instrumental in satisfying”. Individuals usually try to avoid states of isolation, and digital media represent environments to escape from reality (Halfmann and Reinecke, 2021, p. 380) as an emotion-focused-avoidance coping behavior (Knobloch-Westerwick et al., 2009).

Relevantly, TV series on streaming platforms may constitute readily accessible media contents for lonely individuals to escape from feelings of isolation and satisfy the need for social connections, by identifying with a media character, thus experiencing its social relationships within the media narrative. Indeed, previous work has shown that lonely individuals are more prone to emotionally identify with media characters (Greenwood and Long, 2009), and the desire to escape from unpleasant emotions, such as loneliness, is fulfilled more effectively if individuals identify with media characters (e.g., Hefner et al., 2007; Van Looy et al., 2012). The process of identification with media characters is a very well-investigated concept in the media entertainment research (e.g., Oatley, 1994, 1999; Cohen, 2001, 2006) and has been defined as how individuals put themselves in the place of a character and participate in the media character’s experiences. Previous studies have suggested that developing an imaginary relationship with a media persona (Horton and Wohl, 1956) promotes empathic connection with the media character (Green et al., 2004), by experiencing emotional and cognitive absorption into a media narrative. Through this process, viewers temporarily assume the media character’s perspective (Cohen, 2001), and by vicariously experiencing its relationship, viewers satisfy the fundamental human need to develop and maintain meaningful social bonds (e.g., Greenwood, 2008; Greenwood and Long, 2009). In this regard, Baumeister and Leary (1995) suggested that when meaningful relationships are unavailable, individuals may derive partial satisfaction from various substitute interactions, as it could be by identifying with TV series characters.

Building on these assumptions, in the present study we investigated identification, promoted by escapism, as a further distinctive mechanism in the relationship between loneliness and binge-watching behavioral tendencies (Sung et al., 2018).

## Study Overview

The aim of the current study was to investigate specific psychological mechanisms underlying the relationship between loneliness and binge-watching. In particular, considering the association between loneliness and problematic use of digital technologies (e.g., Sung et al., 2018), and building on the assumption that TV series content favor transportation experiences (Wagner, 2016), we hypothesized that only escapism – out of the other factors encompassing the problematic use of Internet-related technologies – would play as a mediator between loneliness and identification with media characters (Hypothesis 1) which in turn would predict greater binge-watching tendencies (Hypothesis 2).

## Materials and Methods

### Participants and Procedures

The study was conducted after receiving the ethical approval from the local commission of the Psychology Department for minimal risk studies. Full informed consent was obtained before participants started the studies. Participants were told that the study would last about 10 to 15 min.

Data were collected through the Prolific web platform using the Qualtrics survey web system during November 2019. As suggested by Prolific guidelines, a “custom whitelist” prescreening was adopted to include specific participants in the study. Prerequisite for participation in the study was to speak English as a primary language. Then, in the prescreening survey, participants were asked to report if they were currently using a TV series/movie streaming service at the time of data collection and watched at least one TV series in the previous seven days. Five hundred and fifty individuals answered the prescreening questionnaire. Six participants were excluded from the analysis because they presented very short response times, while 54 participants reported they had no active streaming service or did not watch any TV series during the 7 days before data collection. Thus, the prescreening sample was composed of 490 individuals from different countries (72.8% United Kingdom; 17.8% United States; 9.4% other).

An *a priori* Monte Carlo power analysis for mediation models (Schoemann et al., 2017) was conducted. The power analysis indicated that considering a small to medium correlation between variables (i.e., *r *= 0.30), a minimum sample size of *N *= 220 was needed to achieve sufficient power based on conventional values (0.80; Cohen, 1988). Therefore, we randomly extracted 220 individuals who received the request to fill in the questionnaire for the main study. To obtain a reliable sample of respondents and identify participants who failed to pay close attention, we included two attentional check items (e.g., “Please answer 3 to this question”; see Oppenheimer et al., 2009). Twenty-one participants failed these checks and were excluded. Thus, the initial sample was composed of 199 participants (121 females, 78 males; *M*_age_ = 33.92 years, *SD *= 9.72; age range 18–60) mainly from the UK (83.7%) and United States (8.9%; other: 7.4%). Participants received € 0.67 for their participation.

### Measures

#### Media Usage

After providing their demographic data, participants were asked to estimate their typical media usage by reporting how many hours per day they usually spend surfing the Internet both for work/study and leisure activities and watching television (on a scale from 0 to 24 h). Afterward, participants were asked which streaming services they had at the time of the data collection, which devices they usually used to watch TV series and report on which day (weekdays/weekend) they usually watched them.

#### Loneliness

The UCLA Loneliness Scale (Russell et al., 1978) was used. The scale is composed of 20 items designed to measure one’s subjective feelings of loneliness as well as feelings of social isolation. One sample item is “I feel left out” (1 = *I never feel this way* to 7 = *I always feel this way*).

#### Problematic Use of Internet-Related Technologies

The Internet Disorder Scale (IDS-15; Pontes and Griffiths, 2017) was adopted for measuring participants’ levels of problematic use of Internet-related technologies. The scale is composed of 15 items (1 = *Very strongly disagree* to 7 = *Very strongly agree*) tapping four distinct latent domains: escapism (e.g., “I go online to help me cope with any bad feelings I might have”), withdrawal symptoms (e.g., “I feel sad if I am not able to go online”), dysfunctional self-regulation (e.g., “I think the amount of time I spend online is negatively impacting on important areas of my life”), and dysfunctional Internet-related self-control (e.g., “I am able to control and/or reduce the time I spend online,” reversely scored).

#### Identification With Media Characters

To measure the identification with the characters of a TV series we adapted the scale proposed by Igartua (2010), composed of 14 items and commonly employed for assessing identifications with media characters. The scale was introduced by asking participants to think about how they usually felt when watching a TV series. One sample item is “I think I am like the characters or very similar to them” (1 = *Very strongly disagree* to 7 = *Very strongly agree*).

#### Binge-Watching Tendency

Participants’ tendency for binge-watching was measured by considering the “Binge-Watching” 6-item subscale of the “Binge-Watching Engagement and Symptoms” scale^1^ proposed by Flayelle et al. (2019). One sample item is “I always need to watch more episodes to feel satisfied” (1 = *Very strongly disagree* to 7 = *Very strongly agree*).

### Results

#### Preliminary and Correlational Analyses

Cronbach’s alphas were all ≥0.83 (see Table 1). Given the adequate internal consistency, we calculated composite scores for each scale, and before conducting the analyses, we inspected data for normality and outliers. A series of multiple regressions were performed with loneliness, the four subscales of the IDS-15^2^ and identification with media characters as the predictor variables and the tendency to binge-watch index as the outcome. Then, we inspected regression diagnostics. Standardized residuals skewness and kurtosis values were all <1.0, indicating a normal distribution of the residuals (Bulmer, 1979). We also tested normality by visually examining the normal Predicted Probability (P–P) plot, finding no drastic deviations from the diagonal. We inspected outliers plotting Cook’s distances by residuals centered leverage (Cook, 1977) for each regression model. Three influential data points emerged, and therefore, we excluded them from all subsequent analyses. Thus, the final sample considered in the analyses described below was composed of 196 participants^3^ (77 males, 119 females; *M*_age_ = 33.76, *SD *= 9.55; age range: 18–60).

**Table 1:** Mean, correlation and reliability for each scale.

		*M*	*SD*	Cronbach’s Alpha	1	2	3	4	5	6	7	8
1	Age	33.76	9.55	–								
2	Gender	–	–	–	0.060							
3	Loneliness	2.88	1.37	*α* = 0.97, 95% CI [0.967, 0.978]	−0.202^**^	−0.081						
4	IDS-15 Escapism	4.17	1.14	*α* = 0.83, 95% CI [0.792, 0.869]	−0.377^**^	−0.086	0.356^**^					
5	IDS-15 Withdrawal symptoms	2.93	1.23	*α* = 0.88, 95% CI [0.859, 0.911]	−0.221^**^	−0.105	0.291^**^	0.509^**^				
6	IDS-15 Dysfunctional self-regulation	3.09	1.23	*α* = 0.86, 95% CI [0.823, 0.888]	−0.304^**^	−0.041	0.239^**^	0.340^**^	0.537^**^			
7	IDS-15 – Dysfunctional self-control	3.41	1.20	*α* = 0.93, 95% CI [0.919, 0.950]	−0.364^**^	−0.108	0.145^*^	0.328^**^	0.453^**^	0.653^**^		
8	Identification with the TV series characters	4.07	0.85	*α* = 0.90, 95% CI [0.880, 0.921]	−0.269^**^	−0.075	0.172^*^	0.359^**^	0.261^**^	0.185^**^	0.087	
9	Binge-watching tendency	3.79	1.22	*α* = 0.89, 95% CI [0.872, 0.917]	−0.261^**^	−0.079	0.211^**^	0.253^**^	0.347^**^	0.341^**^	0.257^**^	0.355^**^

* *p < 0.05*;

** *p < 0.01*.

*N = 196. Participants’ gender was coded as 1 = male, 2 = female. IDS-15 = Internet Disorder Scale*.

At the time of data collection, participants reported to spend, on average, about 5 h per day surfing the Internet for business/school motives (*M *= 4.90, *SD *= 3.06), and about 3 h per day for leisure activities (*M *= 3.18, *SD *= 2.43). They also reported watching television for about 2 h and a half per day (*M *= 2.67, *SD *= 1.69). Furthermore, 8.7% of them reported watching TV series mainly during the weekdays, 5.1% only during the weekend, whereas 86.2% reported both during the weekdays and the weekend. TV series are mainly enjoyed on smart-TVs (83.2%), but also on laptops (33.7%), smartphones (26%), and tablets (24%). In terms of active digital streaming services, participants reported having an active subscription for Netflix (88.3%), Amazon Prime Video (57.7%), Now TV (21.4%), Sky Go (9.2%), Hulu (6.6%), Apple streaming TV (4.1%), Google Play streaming (2%), DisneyLife (1%) or other services (9.1%).

We performed correlational analysis on all the variables. Table 1 summarizes these results.

Loneliness was positively associated with the tendency to binge-watch and with all the four sub-dimensions of the IDS-15 scale. Moreover, escapism, withdrawal symptoms, and dysfunctional self-regulation were also significantly associated with the identification with the TV series characters, which was positively correlated with the tendency to binge-watch. Participants’ age significantly and negatively correlated with all the considered variables; therefore, it was treated as a control variable in the subsequent analyses.

#### Direct and Indirect Effects

To test our hypotheses, namely, that escapism would be a significant mediator of the association between loneliness and identification with TV series characters (Hypothesis 1) that in turn, as a second level mediator, would predict greater binge-watching tendencies (Hypothesis 2) we adopted the PROCESS macro for SPSS (version 3.4; model 80, 5,000 iterations; Hayes, 2017). The tested model considered loneliness as the focal predictor, the four sub-dimensions of problematic Internet-related technologies usage as the first set of mediators and the identification with the TV series characters as the second mediator. The tendency to binge-watch was entered as the outcome variable, whereas age was included as a control variable. The inclusion of all the sub-dimensions of problematic Internet-related technologies usage is functional to provide a tougher test of the efficacy of escapism as a mediator while considering rival parallel mediators.

We evaluated the indirect effects with the joined significance of the components and bootstrap confidence intervals (Yzerbyt et al., 2018). Because of the multiple testing, we corrected the alpha level of the components tests with a Bonferroni correction and adjusted the alpha level at 0.01 (Dunn, 1961), thus computing the 99% confidence intervals.

**Figure 1:**
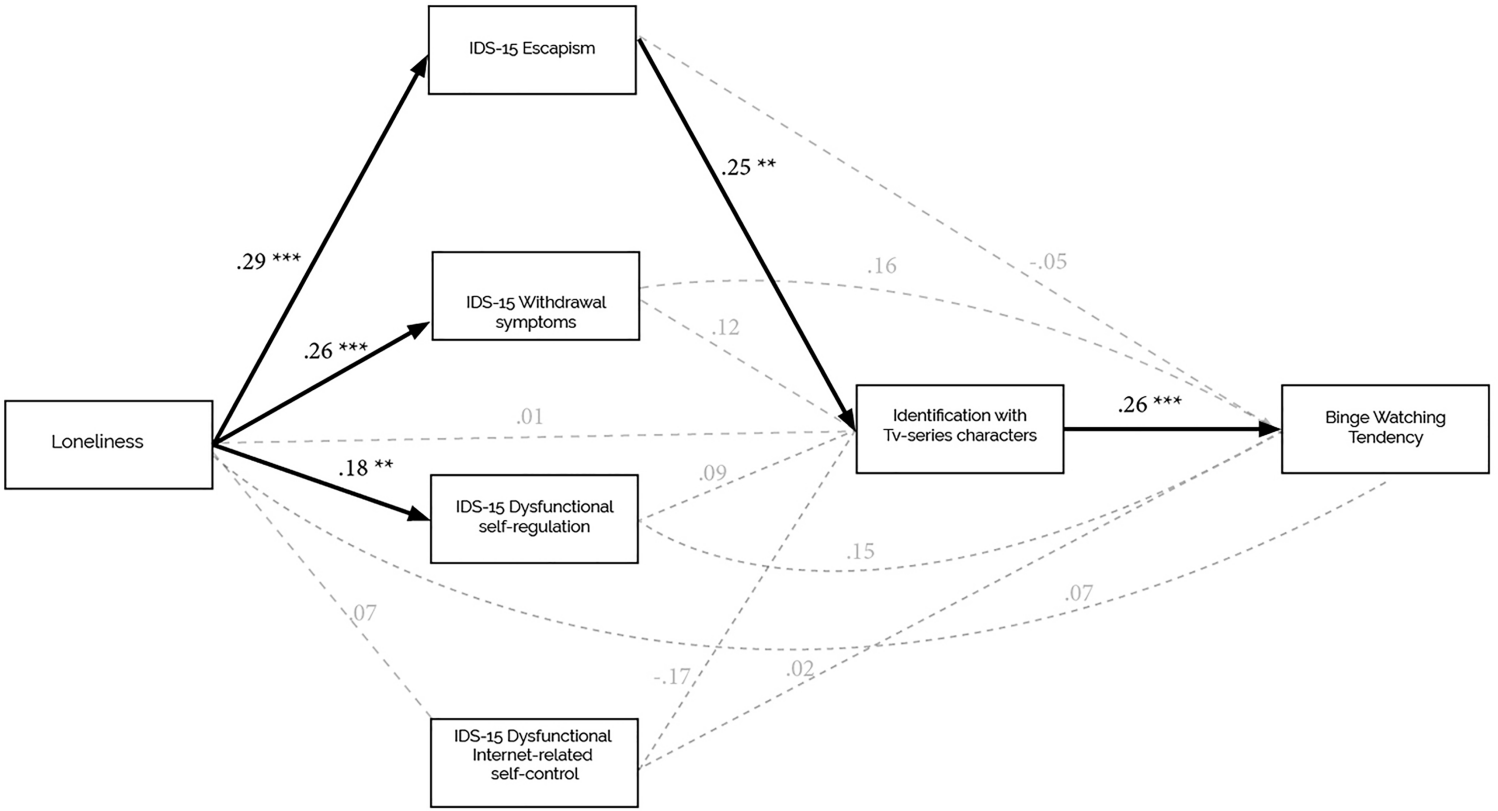
Effects of loneliness on the tendency to binge-watch through the effects of escapism and identification with the media character. Standardized regression coefficients are reported. Bolded arrows represent statistically significant effects; dashed arrows represent non-significant effects. ^**^p < 0.01, ^***^p < 0.001.

As illustrated in Figure 1, our hypotheses were supported: escapism resulted as the unique significant mediator of the link between loneliness and the identification with the TV series characters (Hypothesis 1), that in turn, significantly predicted the tendency to binge-watch (Hypothesis 2). The total amount of variance accounted for by the overall model was *R*^2^ = 0.24. Crucially, the proposed theoretical model was confirmed by the significance of the indirect effect of loneliness through escapism and identification when controlling for age.

Moreover, confirming previous research (e.g., Moretta and Buodo, 2020), results indicated that loneliness was positively associated with withdrawal symptoms, and dysfunctional self-regulation, whereas it was not associated with the dysfunctional Internet-related self-control subscale (see Tables 2 and 3).

**Table 2:** Components, direct effects and total effect.

Predictors	Outcomes	Components and direct effects	*R* ^2^
Loneliness	IDS-15 Escapism	*b* = 0.24, *SE* = 0.05, *β* = 0.29, *t*(193) = 4.51, *p* < 0.001, 99% CI [0.10, 0.38]	0.22
Age		*b* = −0.03, *SE* = 0.01, *β* = −0.32, *t*(193) = −4.92 *p* < 0.001, 99% CI [−0.06, −0.02]	
		
Loneliness	IDS-15 Withdrawal symptoms	*b* = 0.23, *SE* = 0.06, *β* = 0.26, *t*(193) = 3.72, *p* < 0.001, 99% CI [0.07, 0.39]	0.11
Age		*b* = −0.02, *SE* = 0.01, *β* = −0.14, *t*(193) = −2.44, *p* = 0.016, 99% CI [−0.04, 0.001]	
		
Loneliness	IDS-15 Dysfunctional self-regulation	*b* = 0.17, *SE* = 0.06 *β* = 0.18, *t*(193) = 2.70, *p* = 0.007, 99% CI [0.006, 0.32]	0.12
Age		*b* = −0.03, *SE* = 0.01, *β* = −0.26, *t*(193) = −3.88, *p* < 0.001, 99% CI [−0.06, −0.01]	
		
Loneliness	IDS-15 Dysfunctional self-control	*b* = 0.06, *SE* = 0.06, *β* = 0.07, *t*(193) = 1.09, *p* = 0.279, 99% CI [−0.09, 0.22]	0.14
Age		*b* = −0.04, *SE* = 0.01, *β* = −0.35, *t*(193) = −5.11, *p* < 0.001, 99% CI [−0.07, −0.02]	
		
Loneliness	Identification with the TV series characters	*b* = 0.01, *SE* = 0.04, *β* = 0.01, *t*(189) = 0.21, *p* = 0.836, 99% CI [−0.11, 0.12]	0.17
IDS-15 Escapism		*b* = 0.19, *SE* = 0.06, *β* = 0.25, *t*(189) = 3.02, *p* < 0.003, 99% CI [0.03, 0.35]	
IDS-15 Withdrawal symptoms		*b* = 0.08, *SE* = 0.06, *β* = 0.12, *t*(189) = 1.38, *p* = 0.170, 99% CI [−0.07, 0.24]	
IDS-15 Dysfunctional self-regulation		*b* = 0.06, *SE* = 0.06, *β* = 0.09, *t*(189) = 0.99, *p* = 0.324, 99% CI [−0.10 0.23]	
IDS-15 Dysfunctional self-control		*b* = −0.13, *SE* = 0.06, *β* = −0.17, *t*(189) = −1.96, *p* = 0.052, 99% CI [−0.29, 0.04]	
Age		*b* = −0.02, *SE* = 0.01, *β* = −0.18, *t*(189) = −2.42, *p* = 0.016, 99% CI [−0.03, −0.001]	
		
Loneliness	Binge-watching tendency	*b* = 0.07, *SE* = 0.06 *β* = 0.07, *t*(188) = 1.08, *p* = 0.281, 99% CI [−0.09, 0.23]	0.24
IDS-15 Escapism		*b* = −0.05, *SE* = 0.09, *β* = −0.05, *t*(188) = −0.62, *p* = 0.534, 99% CI [−0.28, 0.17]	
IDS-15 Withdrawal symptoms		*b* = 0.16, *SE* = 0.08, *β* = 0.16, *t*(188) = 1.96, *p* = 0.051, 99% CI [−0.05, 0.38]	
IDS-15 Dysfunctional self-regulation		*b* = 0.15, *SE* = 0.09, *β* = 0.15, *t*(188) = 1.70, *p* = 0.090, 99% CI [−0.08, 0.39]	
IDS-15 Dysfunctional self-control		*b* = 0.03, *SE* = 0.09, *β* = 0.02, *t*(188) = 0.29, *p* = 0.772, 99% CI [−0.21, 0.26]	
Identification with the TV series characters		*b* = 0.37, *SE* = 0.10, *β* = 0.26, *t*(188) = 3.68, *p* < 0.001 99% CI [0.11, 0.63]	
Age		*b* = −0.01, *SE* = 0.01, *β* = −0.10, *t*(188) = −1.41, *p* = 0.161, 99% CI [−0.04, 0.01]	
Total Effect		*b* = 0.14, *SE* = 0.06, *β* = 0.16, *t*(193) = 2.36, *p* = 0.019, 99% CI [−0.01, 0.30]	0.09

*N = 196; IDS-15, Internet Disorder Scale*.

**Table 3:** Indirect effects.

Indirect effects	Unstandardized coefficients (Completely standardized coefficients)
Loneliness - > IDS-15 Escapism - > Binge-watching tendency	*IE *= −0.01, 99% CI [−0.08, 0.05] (*IE *= −0.01., 99% CI [−0.09, 0.06])
Loneliness - > IDS-15 Withdrawal symptoms - > Binge-watching tendency	*IE *= 0.04, 99% CI [−0.02, 0.12] (*IE *= 0.04, 99% CI [−0.02, 0.13])
Loneliness - > IDS-15 Dysfunctional self-regulation - > Binge-watching tendency	*IE *= 0.02, 99% CI [−0.02, 0.09] (*IE *= 0.03, 99% CI [−0.02, 0.10])
Loneliness - > IDS-15 Dysfunctional self-control - > Binge-watching tendency	*IE *= 0.00, 99% CI [−0.02, 0.04] (*IE *= 0.00, 99% CI [−0.03, 0.04])
Loneliness - > Identification with the TV series characters - > Binge-watching tendency	*IE *= 0.00, 99% CI [−0.04, 0.04] (*IE *= 0.00, 99% CI [−0.05, 0.05])
**Loneliness - > IDS-15 Escapism - > Identification with the TV series characters - > Binge-watching tendency**	***IE *= 0.02, 99% CI [0.001, 0.05] (*IE *= 0.01, 99% CI [0.002, 0.05])**
Loneliness - > IDS-15 Withdrawal symptoms - > Identification with the TV series characters - > Binge-watching tendency	*IE *= 0.00, 99% CI [−0.01, 0.02] (*IE *= 0.00., 99% CI [−0.01, 0.02])
Loneliness - > IDS-15 Dysfunctional self-regulation - > Identification with the TV series characters - > Binge-watching tendency	*IE *= 0.00, 99% CI [−0.01, 0.02] (*IE *= 0.00., 99% CI [−0.009, 0.02])
Loneliness - > IDS-15 Dysfunctional self-control - > Identification with the TV series characters - > Binge-watching tendency	*IE *= −0.00, 99% CI [−0.02, 0.005] (*IE *= −0.00, 99% CI [−0.02, 0.005])

*N = 196; IE, indirect effect; IDS-15, Internet Disorder Scale. Significant effects are in bold*.

## Discussion

During the last decade, the advent of fast network connections has facilitated the spread of media streaming platforms, offering easy access to a large amount of digital media content. In this regard, Netflix has promoted the idea that binge-watching represents a normalized, new means of television viewing facilitated by up-to-date and current technological advancements. The binge-watching phenomenon is relatively new, and research in recent years has mainly focused on the motivations (e.g., Pittman and Sheehan, 2015; Panda and Pandey, 2017; Sung et al., 2018) and the negative outcomes of this behavior (e.g., De Feijter et al., 2016; Exelmans and Van den Bulck, 2017; Spruance et al., 2017).

In the present work we, instead, focused on some of the possible mechanisms underlying binge-watching, showing that loneliness is one determinant of this behavior, through the desire to escape from reality, followed by the identification with media characters. Our results replicated previous studies (see Tokunaga and Rains, 2010, for a review), suggesting that higher feelings of loneliness are associated with higher levels of problematic use of digital technologies. Nevertheless, considering the specific domain of TV series, direct and indirect effects suggest that only escapism significantly predicts binge-watching tendencies, through the mediating effect of identification with media characters. Given the highlighted links, we speculate that an excessive consumption of streaming TV series could be interpreted as a behavioral coping strategy for media escapists. It is worth noticing that using TV series as a means of “escape” is not necessarily problematic. In fact, escape motives are among the most significant reasons for using entertainment media, such as television (Vorderer and Klimmt, 2021). Nevertheless, individuals may adopt binge-watching behaviors as an effective coping strategy because it requires low effort (Perks, 2019): People whose energy reserves are depleted may be more likely to stumble into problematic binge-watching because of the undemanding nature of the activity. We suggest that binge-watching, due to the entertaining nature and ease-of-access of digital streaming services, allows lonely people to satisfy the need for escapism as a coping strategy to compensate for the lack of social ties, by identifying with a fictional character.

In fact, binge-watching is an activity that, once started, is easy to continue: when approaching the end of an episode, the next one is immediately queued to play, meaning that it is actually more demanding to stop than to continue viewing the show (Pittman and Sheehan, 2015). Furthermore, continually watching a TV series featuring similar characters, plots, and locations is cognitively less demanding than switching to other activities, favoring problematic media consumption behavior.

It is worth mentioning that participants’ age also emerged as a significant covariate in the tested model. This is not surprising since at-risk digital use has been found to be strongly dependent on age (e.g., Bakken et al., 2009). Furthermore, age was negatively associated with loneliness. This result is in line with previous evidence, suggesting that adolescence is the peak age for experiencing loneliness (see Yang and Victor, 2011, for a review).

## Limitations and Future Research Directions

Although this study provides important new insights, because of its correlational design, no conclusions about causality can be drawn. It could be that loneliness and binge-watching feed one another in a reciprocal way; namely, the more individuals experience loneliness, the more they binge-watch as a coping strategy, which in turn leads them to greater feelings of loneliness because what causes those feelings has not been addressed properly. Longitudinal and experimental studies should be designed to help uncover causal links.

A second limitation of the present study is that we did not consider individual differences, which may offer a further explanation of the mechanisms underlying binge-watching behaviors. The proposed model explained 24% of the variance in binge-watching tendency, suggesting that the considered predictors play an important role, but there is still much to uncover. Likely, including individual differences in the investigation would increase our understanding of binge-watching. In this regard, the need for cognition (Cacioppo and Petty, 1982) and the need for affect (Maio and Esses, 2001) may represent significant moderators of identification with a media character. Indeed, both these needs play a significant role in social perception (Aquino et al., 2016), and the need for affect has been found to be a relevant predictor of narrative transportation (Appel and Richter, 2010). Thus, it is plausible to hypothesize that both individual characteristics could moderate the impact of identification with different kinds of media characters (e.g., cognitively vs. emotional complex characters, respectively).

Moreover, previous literature agrees on the importance of deepening the role of the time spent using Internet-related technologies when investigating digital addictions. In this regard, on the one hand, Triberti et al. (2018) suggested that the time spent playing online video games is not important in an absolute sense, but relatively to specific day phases, meaning that intense use may not be indicative of problematic use *per se*. On the other hand, other studies suggest that the intense and prolonged use of social media platforms over time may foster greater symptoms of addiction (see Leong et al., 2019).

Thus, longitudinal studies aimed at exploring the specific course of binge-watching over time should be encouraged. Such studies could be of primary importance to distinguish situations in which binge-watching is a temporary strategy adopted by individuals for dealing with difficult circumstances (e.g., different levels of perceived loneliness across time), from other cases where binge-watching is a consequence of more persistent psychological conditions (e.g., depression), and to identify its different mediators and moderators. Longitudinal studies could also allow researchers to verify the actual interference that Internet-related addictive conducts can have on other relevant everyday activities (see, e.g., Young, 2004).

A third limitation of the present study is the operationalization of the binge-watching phenomenon. Although we were interested in understanding what mechanisms facilitate this behavior and not the long-term positive or negative consequences of it, future studies should adopt more stringent measures of TV series consumption. As said, a shared definition and operationalization of the phenomenon is still under debate. Moreover, binge-watching can be beneficial in many instances as a stress-reliever or for bonding with other people by discussing common topics (such as the plot of a popular TV series). Thus, to better understand the impact that this behavior may have on individuals’ well-being, it is important to reach an agreed definition of binge-watching to favor the development of on-point measurements (Flayelle et al., 2019).

Early theoretical insights (Griffiths, 1998, 2000) suggested that lonely people use digital technology to cope with their situation. In the present work, we indeed propose that the tendency to binge-watch could be interpreted as a coping strategy that allows lonely people to escape from their unpleasant emotions through identification with a fictitious character. This behavior could be part of a broader problematic use of Internet-related technologies. Future research should determine whether binge-watching is associated with problematic behaviors related to other similar media products, such as video games or online role-playing games, that could enable the same identification mechanisms and coping strategies. In this regard, other studies might focus on the technological differences underlying the different streaming platforms. For example, some platforms propose the automatic viewing of the next episode within 15 s, others within 30 s. Indeed, most of the streaming platforms offer the possibility to turn off the autoplay of the next episode which, however, is usually the default option. It is therefore plausible to assume that the tendency to binge-watch could be limited simply by disabling this feature.

## Conclusion

Modern online technologies represent a valuable tool to support work activities and facilitate communication between people, but they can also be entertaining, and we cannot – nor should – avoid using such technologies. Indeed, we believe that modern digital technologies should be designed to be life-enriching. Future research should take a more holistic approach to understand how new digital technologies affect our lives, encouraging the design of digital tools helping people to cope with negative emotions and avoiding to “watch the next episode” to deal with their problems.

## Data Availability Statement

The raw data supporting the conclusions of this article will be made available by the authors on request, without undue reservation.

## Ethics Statement

The studies involving human participants were reviewed and approved by Local commission of the Psychology Department (University of Milano Bicocca) for minimal risk studies. The patients/participants provided their written informed consent to participate in this study.

## Author Contributions

AG and CB contributed to the conception and the design of the work. AG was responsible for the data collection and wrote the manuscript with valuable inputs from the remaining authors. AG and CB were responsible for the analysis. All the authors contributed to the interpretation of data and agreed for all aspects of the work and approved the version to be published.

## Conflict of Interest

The authors declare that the research was conducted in the absence of any commercial or financial relationships that could be construed as a potential conflict of interest.

## Publisher’s Note

All claims expressed in this article are solely those of the authors and do not necessarily represent those of their affiliated organizations, or those of the publisher, the editors and the reviewers. Any product that may be evaluated in this article, or claim that may be made by its manufacturer, is not guaranteed or endorsed by the publisher.

## Footnotes

^1^The original scale taps seven distinct factors for assessing motives and correlates for binge-watching intended both as a personal enrichment and as a coping strategy. We choose to use only the binge-watching subscale because the other factors overlapped with the dimensions measured by the IDS-15 (i.e., dependency and loss of control) or because they were not relevant for the present work (i.e., engagement, desire/savoring, positive emotions, and pleasure preservation).

^2^An Exploratory Factor Analysis (EFA) was conducted on the 15 items of the IDS-15 scale. Maximum likelihood extraction method was used in combination with an Oblimin rotation. The Kaiser-Meyer-Olkin was=.88, which is well above the recommended threshold of 0.6 (Kaiser, 1974), and all KMO values for individual items were > 0.84, all above the acceptable limit of 0.5 (Field, 2009). Bartlett’s test of sphericity, *χ*^2^ (105) = 2013, *p* < 0.001, indicated that correlations between items were sufficiently large for EFA. The number of factors was extracted based on parallel analysis. The analyses confirmed the 4-factor structure of the scale, explaining 67.8% of the variance.

^3^Since the sample size suggested by the a priori power analysis was not reached, a post hoc Monte Carlo power analysis (Schoemann et al., 2017) considering the obtained correlations between our focal predictors (i.e., loneliness, escapism, identification, and binge-watching tendency), their standard deviations, 1000 replications, 20.000 draws per replication and random seed = 1234 and confidence level =99%, was conducted. The power analysis indicated that, considering our sample size, the achieved power for the considered indirect effect was equal to 0.92 (but see also Lakens, 2021).
